# Cyclic RGD-Functionalized pH/ROS Dual-Responsive Nanoparticle for Targeted Breast Cancer Therapy

**DOI:** 10.3390/pharmaceutics15071827

**Published:** 2023-06-26

**Authors:** Pu Yao, Xiaowen Wang, Qianmei Wang, Qing Dai, Yu Peng, Qian Yuan, Nan Mou, Shan Lv, Bangbi Weng, Yu Wang, Fengjun Sun

**Affiliations:** 1 Department of Pharmacy, Southwest Hospital, Army Medical University (Third Military Medical University), Chongqing 400038, China; 2 Department of Oncology, Southwest Hospital, Army Medical University (Third Military Medical University), Chongqing 400038, China

**Keywords:** pH/ROS dual-responsive NPs, drug release, cRGD, breast cancer, cancer therapy

## Abstract

Breast cancer is the most common malignant tumor in women and is a big challenge to clinical treatment due to the high morbidity and mortality. The pH/ROS dual-responsive nanoplatforms may be an effective way to significantly improve the therapeutic efficacy of breast cancer. Herein, we report a docetaxel (DTX)-loaded pH/ROS-responsive NP that could achieve active targeting of cancer cells and selective and complete drug release for effective drug delivery. The pH/ROS-responsive NPs were fabricated using nanocarriers that consist of an ROS-responsive moiety (4-hydroxymethylphenylboronic acid pinacol ester, HPAP), cinnamaldehyde (CA, an aldehyde organic compound with anticancer activities) and cyclodextrin (α-CD). The NPs were loaded with DTX, modified with a tumor-penetration peptide (circular RGD, cRGD) and named DTX/RGD NPs. The cRGD could promote DTX/RGD NPs penetration into deep tumor tissue and specifically target cancer cells. After internalization by cancer cells through receptor-mediated endocytosis, the pH-responsive acetal was cleaved to release CA in the lysosomal acidic environment. Meanwhile, the high ROS in tumor cells induced the disassembly of NPs with complete release of DTX. In vitro cellular assays verified that DTX/RGD NPs could be effectively internalized by 4T1 cells, obviously inducing apoptosis, blocking the cell cycle of 4T1 cells and consequently, killing tumor cells. In vivo animal experiments demonstrated that the NPs could target to the tumor sites and significantly inhibit the tumor growth in 4T1 breast cancer mice. Both in vitro and in vivo investigations demonstrated that DTX/RGD NPs could significantly improve the antitumor effect compared to free DTX. Thus, the DTX/RGD NPs provide a promising strategy for enhancing drug delivery and cancer therapy.

## 1. Introduction

Breast cancer, as the most commonly malignant cancer in the world, remains the greatest challenge in clinical cancer research [1,2,3]. Clinically, the current treatment strategies include surgery, chemotherapy, radiation therapy and hormonotherapy [4]. Of these, chemotherapy is the main treatment method for breast cancer. Nevertheless, most chemotherapy drugs are poorly water-soluble, which is not conducive to intravenous administration [5] Other adverse characteristics of chemotherapy drugs, such as low stability, systemic toxicity, drug resistance and insufficient drug concentration at the tumor site have also limited its further application [5,6]. A great deal of nanomedicines have been developed for antitumor therapy, which can overcome the biological barriers and deliver chemotherapy drugs into tumors. Compared with conventional chemotherapy, nanomedicines are able to change the pharmacokinetics, improve the stability and solubility of small-molecule chemotherapeutic drugs and accumulate at target sites, thereby reducing the side effects of chemotherapy and improving the antitumor effect [7,8,9,10,11]. However, due to the physiological characteristics of the body and the heterogeneity of the tumor, the pharmacological effect of nanomedicines also needs to be improved [12]. Recently, the stimuli-responsive nanomedicines have drawn extensive attention due to their smart release and enhanced therapy, which would address challenges and limitations in the clinic [13,14,15]. These stimuli-responsive nanomedicines respond to external stimuli (e.g., light, ultrasound, magnetic field, etc.) [16,17] or internal abnormal physiological stimuli (e.g., pH, enzymes, ROS, etc.) [18,19,20] for targeted delivery and release of encapsulated therapeutic agents. These nanomedicines have greatly increased the drug concentration at tumor sites and achieved controlled release of the drug at tumor sites, reducing the toxicity and side effects.

As one of the characteristics of the tumor microenvironment, a high concentration of ROS has been widely used in the design of nanomedicines [21]. Although the concentration of ROS in breast cancer cells is much higher than that in normal cells (10–50 times) [22,23], it is still difficult to fully release the anticancer drugs loaded on the nano-drug-delivery systems by solely relying on the intrinsic ROS of tumor cells [24,25,26]. In addition, tumor cells can also reduce excessive ROS by upregulating antioxidants such as glutathione (GSH) to maintain intracellular redox homeostasis, further weakening the effectiveness of ROS-responsive drug-delivery system [27]. Otherwise, pH-responsive nanocarriers have been considered to be one of the most promising systems for intracellular delivery of various therapeutics in acidic endosomal/lysosomal compartments (pH 4–5) [28], but the complete release of the drug may also be limited with the H^+^ exhausted without adequate supplementation. Taken together, pH/ROS dual-responsive nanoplatforms may be an effective way to significantly enhance selective and complete drug release, as well as improve the therapeutic efficacy against breast cancer.

However, this passive targeting strategy cannot ensure an optimal accumulation of drugs in the tumor and prevent undesirable side effects simultaneously [29,30]. The ability of nanomedicines to recognize tumor sites must be improved. Numerous studies have shown that the expression of integrin αvβ3 is significantly increased in tumor blood vessels and various invasive tumor cells, while in normal resting endothelial cells and tissues, the expression level is relatively low and almost non-expressive, making it an ideal target for tumor therapy [31,32]. The RGD peptide (Arginine-Glycine-Aspartic) can specifically bind to integrins, with especially strong binding to αvβ3. Moreover, not only is it an ideal tumor target factor, but it can also directly induce tumor apoptosis by triggering the activation of caspase-3 [33]. A large number of studies has shown that cRGD has a significant inhibitory effect on angiogenesis that is superior to the linear RGD peptide [34]. Due to the αvβ3 and cRGD peptides having a high affinity, cRGD-modified nanoparticles can bind to αvβ3 integrins on the tumor endothelium to penetrate tumor cells and achieve a satisfactory therapeutic effect [35,36,37]. Nanoparticles modified with cRGD are internalized by cancer cells through receptor-mediated endocytosis and are transported to lysosomes [38]. The lysosomal acidic environment (pH 4.5–6.0) provides an advantage for designing pH-responsive nanocarriers [39,40].

**Figure 1:**
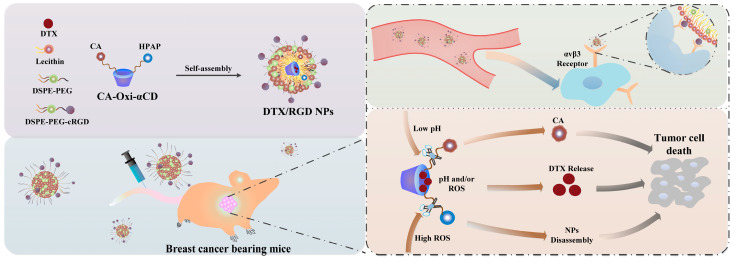
Fabrication of DTX-loaded pH/ROS dual-responsive NPs for targeted breast cancer therapy.

In this study, we developed a cRGD-functionalized pH/ROS dual-responsive nanocarrier to load DTX, a famous chemotherapeutic agent against breast cancer. The nanocarrier consists of an ROS-responsive moiety (HPAP), CA (an aldehyde organic compound with anticancer activities) [41] and α-CD (Scheme S1). Using this nanocarrier, DTX-loaded pH/ROS dual-responsive NPs were prepared using a nanoprecipitation/self-assembly method. This NP, named DTX/RGD NP, is modified by a tumor-tissue-penetration and cancer-cell-targeting peptide (cRGD) on the surface. The surface-modified cRGD could facilitate the DTX/RGD NPs’ penetration into deep tumor tissue and specifically target cancer cells. After internalization by cancer cells, in the lysosomal acidic environment, the acetal between CA and α-CD in the nanocarriers is cleaved to release CA. Meanwhile, the intrinsic ROS can attack the boron atom in the HPAP group as a nucleophile, resulting in the oxidation and breakage of borate ester, followed by the electron transfer process. Then the chemical bonding between α-CD and the phenyl group is broken to induce the disassembly of NP to completely release DTX, consequently killing tumor cells and enhancing cancer therapy (Figure 1).

## 2. Materials and Methods

### 2.1. Materials

Pluronic F-127 (polyethylene-polypropylene glycol) were purchased from Sigma–Aldrich Co. (Shanghai, China). Cy5 free acid was provided by Lumiprobe, LLC. (Hallandale Beach, FL, USA). DSPE-PEG_2000_, DSPE-PEG-cRGD (cyclic Arg-Gly-Asp) and polystyrene nanoparticles (PS NPs) were obtained from Xi’an Ruixi Biotechnology Co., Ltd. (Xi’an, China). The Dulbecco’s modified Eagle’s medium (DMEM) and fetal bovine serum were provided by HyClone Inc. (Waltham, MA, USA). The streptomycin–penicillin solution and FITC-phalloidin were obtained from Solarbio Life Sciences Co., Ltd. (Beijing, China). The 4′,6-Diamidino-2-phenylindole (DAPI), Enhanced Cell Counting Kit-8 (CCK-8), Hydrogen Peroxide Assay Kit and Cell Cycle Kit were supplied by Beyotime Biotechnology Co., Ltd. (Shanghai, China). The Matrigel was purchased from Corning Inc. (Corning, NY, USA). An Annexin V-FITC Apoptosis Detection Kit was provided by Becton, Dickinson and Company (Franklin Lakes, NJ, USA).

### 2.2. Cell Culture

The mouse breast cancer cell line 4T1 and human breast cancer cell line MDA-MB-231 were provided by the Cell Bank of the Chinese Academy of Sciences (Shanghai, China). The cells were cultured in a DMEM cell culture medium supplemented with 10% fetal bovine serum, 100 µg/mL streptomycin and 100 IU penicillin at 37 °C in a humidified atmosphere containing 5% CO_2_. The cell culture medium was changed every two days and cells were passaged in a 1:3 ratio.

### 2.3. Animals

Six- or eight-week-old female BAlB/c mice weighing approximately 20 g were supplied from the experimental animal center of Army Medical University (Chongqing, China) and kept in an SPF-level sterile animal room. All animal experiments were performed in accordance with the guidelines approved by the ethics committee of the Army Medical University (Chongqing, China).

### 2.4. Fabrication and Characterization of DTX-Loaded NPs

The DTX-loaded NPs were fabricated using a nanoprecipitation/self-assembly method. Briefly, 6 mg of lecithin and 6 mg of DSPE-PEG_2000_ were dispersed in 400 µL of anhydrous ethanol and 7.0 mL of deionized water. The dispersed solution was heated at 65 °C for 0.5 h. Meanwhile, 50 mg of CA-Oxi-αCD and 10 mg of DTX were dissolved in 400 µL of methanol and 200 µL of DMSO, then the drug-containing solution was added dropwise to the lipid-dispersed solution with gentle stirring followed by fast stirring for 3 min. After self-assembly for 2 h under slow stirring at room temperature, the DTX-loaded NPs were harvested and centrifuged at 15,000× *g* for 10 min with a high-speed freezing centrifuge (Thermo Scientific Inc., Waltham, MA, USA). After being washed with 5% F127 twice, the NPs were then suspended in ultrapure water. Additionally, the RGD-modified DTX-loaded NPs were fabricated using a similar procedure as the NPs with slight changes: 4 mg of DSPE-PEG_2000_ and 4 mg of DSPE-PEG-cRGD were added to the lipid dispersion to fabricate the NPs. Furthermore, 5 mg of Cy5-conjugated α-CD was added to fabricate the Cy5-labeled NPs. The particle size, polydispersity index (PDI) and zeta potential of the NPs were determined with dynamic light scattering (DLS) and laser Doppler anemometry using a Malvern Zetasizer (Nano ZS, Malvern, UK). The morphology of the NPs was observed with a transmission electron microscope (TEM, JEM-1400, JEOL Ltd., Tokyo, Japan). The drug loading and encapsulation efficiency were determined as previously reported [42].

### 2.5. pH/ROS Responsiveness and Drug Release of NPs In Vitro

To investigate the responsiveness of DTX-loaded NPs under H_2_O_2_/pH conditions, the NPs were dispersed in H_2_O, pH 5.0, 1 mM H_2_O_2_ or pH 5.0/1 mM H_2_O_2_ medium for 2 h. Then the size changes of the NPs under various conditions were determined using Malvern Zetasizer (Nano ZS, Malvern, UK).

The in vitro release behavior of DTX and CA from the NPs was performed using a dialysis method. Briefly, 200 μL of the NPs was added into a dialysis bag (MWCO: 3500 Da), then the dialysis bag containing the NPs was immersed into 40 mL of PBS, 1 mM H_2_O_2_ in PBS, PBS at pH 5.0 or 1 mM H_2_O_2_ in PBS at pH 5.0 with 1% (*w*/*v*) Tween 80. Following gently shaking at a speed of 100 rpm at 37 °C, 4.0 mL of external release medium was collected at the determined time and replaced with 4.0 mL of fresh medium at the same time. The concentrations of DTX and CA at 0.08, 0.25, 0.5, 1, 2, 4, 6, 8, 10, 12, 24 and 48 h were detected using high-performance liquid chromatography (HPLC, Waters, Milford, MA, USA), and the cumulative drug-release percentage was calculated accordingly.

### 2.6. Hemolysis Assay

The biocompatibility of the NPs with sheep blood cells was evaluated with a hemolysis assay. Briefly, NPs were incubated with 3% red blood cell suspensions at different concentrations (19.6, 39.2, 78.4, 156.8, 313.6, 627.2 μg/mL) for 1 h at 37 °C in 5% CO_2_. PBS was used as the negative control, and the positive control was a 1% *w*/*v* solution of Triton X-100. The cells were then centrifuged at 3000 rpm for 5 min, the supernatants were carefully collected and the absorbance of the supernatants was detected with a Thermo Multiskan Spectrum spectrophotometer (Thermo Scientific Inc., Waltham, MA, USA) at a wavelength of 450 nm. The hemolytic percentage (hemolysis %) was calculated according to the following equation: Hemolysis % = [A_450_ (NPs) − A_450_ (PBS)]/[A_450_ (1% Triton X-100) − A_450_ (PBS)] × 100%.

### 2.7. Intracellular H_2_O_2_ Detection

4T1 cells were seeded at a density of 2 × 10^5^ cells per well in 12-well plates and allowed to grow for 24 h at 37 °C in 5% CO_2_. Then the cells were treated with DTX, PS NPs, DTX/NPs and DTX/RGD NPs with an equivalent concentration of 5 ng/mL of DTX in cell culture medium for 48 h at 37 °C in 5% CO_2_. The control was treated with cell culture medium. After 48 h of incubation, the intracellular concentrations of H_2_O_2_ in 4T1 cells were determined according to the manufacturer’s protocols of a Hydrogen Peroxide Assay Kit.

### 2.8. Intracellular Uptake

4T1 cells were cultured in confocal dishes for 24 h at 37 °C in 5% CO_2_. The cells were then treated with free Cy5, Cy5-labeled NPs or Cy5-labeled RGD NPs with an equivalent concentration of 2 µg/mL of Cy5 for 4 h at 37 °C in 5% CO_2_. Then the cells were washed and fixed with 4% paraformaldehyde for 20 min. After being permeabilized with 0.5% Triton-X for 5 min at room temperature, the cells were incubated with FITC-phalloidin (100 nM) for 30 min at 37 °C in the dark. Then the cell nuclei were stained with DAPI (5 μg/mL) for 10 min at room temperature, and the intracellular uptake of the NPs was obtained with confocal laser scanning microscopy (CLSM, Carl Zeiss, Baden-Württemberg, Germany).

We also quantitatively analyzed the uptake of NPs in 4T1 cells with flow cytometry. Briefly, 4T1 cells were seeded at a density of 2 × 10^5^ cells per well in 12-well plates and cultured for 24 h at 37 °C in 5% CO_2_. Then the cells were incubated with free Cy5, Cy5-labeled NPs or Cy5-labeled RGD NPs with an equivalent concentration of 2 µg/mL of Cy5 for 4 h at 37 °C in 5% CO_2_. After incubation, the cells were collected and the fluorescence intensity of intracellular Cy5 was detected using flow cytometry (Accuri C6, BD, Franklin Lakes, NJ, USA).

### 2.9. The Penetration in 3D Tumor Spheroids

4T1 cells were seeded on confocal dishes precoated with a thin layer of Matrigel. Then the cells in the tumor spheroids were incubated with Cy5, Cy5-labeled NPs or Cy5-labeled RGD NPs with an equivalent concentration of 2 µg/mL of Cy5 for 4 h at 37 °C in 5% CO_2_. After the cells were washed with cold PBS three times and fixed in 4% paraformaldehyde for 20 min at room temperature, the cell nuclei were stained with DAPI (5 μg/mL) for 10 min at room temperature. Finally, the penetration of the NPs in the 3D tumor spheroids was observed using CLSM (Carl Zeiss, Baden-Württemberg, Germany) in layer-scanning mode.

### 2.10. Cytotoxicity Assays

The in vitro cytotoxicity of NPs against 4T1 cells was evaluated using the CCK-8 assay. 4T1 cells were seeded in 96-well plates at 1 × 10^4^ cells per well for 24 h at 37 °C in 5% CO_2_. Then the cells were treated with DTX, blank NPs or DTX-loaded NPs at a concentration of 0.31, 1.25, 5.00, 20.00 or 80.00 ng/mL for 24 h or 48 h at 37 °C in 5% CO_2_. After treatment, the cells were washed with PBS three times and incubated with CCK-8 solution for another 0.5 h at 37 °C in 5% CO_2_. Finally, the absorbance of the cultures was measured at 450 nm using a Thermo Multiskan Spectrum spectrophotometer (Thermo Scientific Inc., Waltham, MA, USA). The 50% inhibitory concentration (IC_50_) was calculated by plotting the dose–response curve using GraphPad Prism 8.0.

### 2.11. Cell Cycle Assay

To investigate the effect of NPs on the tumor cell cycle, 4T1 cells were cultured in 12-well plates at a density of 2 × 10^5^ cells per well for 24 h at 37 °C in 5% CO_2_. The cells were treated with DTX, blank NPs, DTX/NPs or DTX/RGD NPs with an equivalent concentration of 20 ng/mL of DTX in cell medium at 37 °C in 5% CO_2_ for 48 h. After 48 h of incubation, the cells were centrifuged and washed with cold PBS three times. Then the cells were fixed with cold 70% ethanol for 24 h at 4 °C. Finally, the cells were stained with PI according to the manufacturer’s instructions and determined with flow cytometry (Accuri C6, BD, Franklin Lakes, NJ, USA).

### 2.12. Cell Apoptosis Assay

4T1 cells were seeded in 12-well plates at a density of 2 × 10^5^ cells per well and allowed to grow for 24 h at 37 °C in 5% CO_2_. The cells were then incubated with DTX, blank NPs, DTX/NPs or DTX/RGD NPs with an equivalent concentration of 20 ng/mL of DTX in cell medium at 37 °C in 5% CO_2_ for 48 h. After 48 h of incubation, the cells were collected and the apoptosis percentage was determined with an Annexin V-FITC apoptosis-detection kit according to the manufacturer’s instructions.

### 2.13. In Vivo Biodistribution

We investigated the in vivo distribution of NPs in mice bearing a 4T1 breast cancer model. Briefly, the mice were injected with 1 × 10^6^ 4T1 cells into the fourth mammary fat pad (right). When the tumor volume grew to approximately 300 mm^3^, the mice were injected with Cy5, Cy5-labeled NPs or Cy5-labeled RGD NPs at a Cy5 dosage of 1 mg/kg via vein. After administration for 2 h, 8 h, 24 h or 48 h, the mice were anesthetized and imaged with an in vivo imaging system (Perkin Elmer, Waltham, MA, USA). At 48 h post-injection, the mice were sacrificed and the tumors and major tissues were harvested and imaged. The semi-quantitative analysis of fluorescence intensity in tumors was obtained through an in vivo imaging system software. In addition, the cryosectioned tumor tissues were fixed with 4% paraformaldehyde for 20 min, and the nuclei were then stained with DAPI (5 μg/mL) for 10 min at room temperature and finally imaged with CLSM (Carl Zeiss, Baden-Württemberg, Germany).

### 2.14. Antitumor Efficacy In Vivo

To investigate the antitumor efficacy of DTX/RGD NPs in vivo, the in vivo 4T1 breast tumor model was established. The female BALB/c mice were subcutaneously injected with 5 × 10^5^ 4T1 cells into the fourth mammary fat pad. Five days after inoculation, tumor-bearing mice were randomly divided into 5 groups (*n* = 6) and treated with saline, blank NPs, DTX, PLGA NPs or DTX/RGD NPs via vein at a dose of 5 mg/kg DTX every four days for five times. The tumor volumes and body weights were measured every 2 days. The tumor volumes of the mice were measured and calculated using the following formula: V = 1/2 (L × W^2^), in which L (length) is the longest diameter and W (width) is the shortest diameter perpendicular to length. After the termination of the therapy, all mice were sacrificed, and the tumors and lungs were collected for photo imaging, weighing and histopathologic examination. The remaining tumors were homogenized, and the DTX concentration in the tumors was determined using LC-MS/MS (AB SCIEX Qtrap5500, Shimadzu, Kyoto, Japan).

### 2.15. Statistical Analysis

All data are reported as the mean ± standard deviation (SD) with at least three independent experiments. Statistical analysis was performed using the unpaired two-tailed Student’s *t*-test for two groups and one-way analysis of variance (ANOVA) with Tukey’s multiple comparisons test for multiple-group comparisons. Statistical significance was defined as * *p* < 0.05, ** *p* < 0.01, *** *p* < 0.001, **** *p* < 0.0001.

## 3. Results and Discussion

### 3.1. Fabrication and Characterization of the NPs

**Figure 2:**
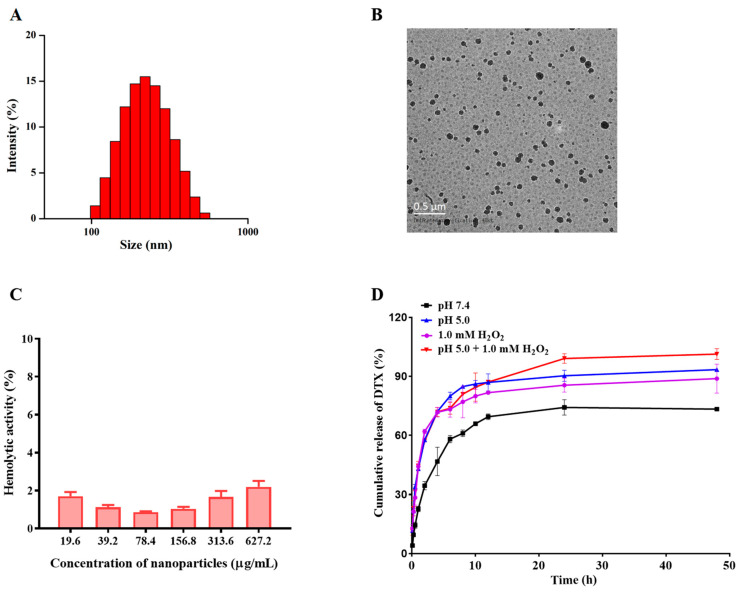
Characterization of DTX/RGD NPs. (**A**) The size distribution of NPs determined with DLS. (**B**) The morphology of NPs observed with TEM. Scale bar represents 0.5 μm. (**C**) The hemolytic activity of NPs at various concentrations. (**D**) The in vitro drug-release behavior of DTX from NPs in various release media within 48 h. Each value represents the mean ± SD (*n* = 3).

The detailed synthetic route and the characterization of the pH/ROS dual-responsive materials (CA-Oxi-αCD) are displayed in the Supporting Information (Scheme S1, Figure S1). By using the CA-Oxi-αCD materials as the carrier, the DTX/RGD NPs were fabricated using a nanoprecipitation/self-assembly method. As shown in Table S1 and Figure 2A, the average diameter of the DTX/RGD NPs was 217.00 ± 2.70 nm with good dispersity and the zeta potential was −19.37 ± 0.58 mV. The morphology of the DTX/RGD NPs was imaged using TEM. The TEM images showed that the DTX/RGD NPs had a spherical morphology with a good distribution (Figure 2B). Furthermore, the particle size observed in the TEM was generally consistent with that measured using DLS. These NPs also displayed a satisfactory drug loading and encapsulation efficiency. As listed in Table S1, the DTX loading and encapsulation efficiency of the DTX/RGD NPs were 19.37 ± 3.05% and 73.32 ± 5.04%, respectively. Moreover, the NPs exhibited good blood compatibility (Figure 2C).

The size changes of DTX-loaded NPs in acidic and/or ROS conditions were determined using DLS to evaluate the capability of the pH/ROS dual-responsiveness. As shown in Figure S2, after incubation in 1.0 mM H_2_O_2_ for 2 h, the size of NPs was changed from 261.10 ± 6.72 nm to 151.97 ± 1.76 nm, and the PDI of NPs was changed from 0.18 ± 0.03 to 0.06 ± 0.02, owing to ROS-triggered degradation of HPAP in the carrier and subsequent dissociation of the NPs, resulting in the promotion of drug release. Moreover, the changes in size of the NPs in the pH 5.0/1.0 mM H_2_O_2_ medium was similar to the 1.0 mM H_2_O_2_ medium. Additionally, the size of the NPs at pH 5.0 medium decreased about 50 nm compared to the NPs distributed in water, implying that an acidic pH might cause the disintegration of the NPs construction, which helps to release the drug and diffuse it towards the tumors’ core [43]. However, the PDI of the NPs after incubation in pH 5.0 medium showed no significant changes.

The drug-release behavior of the DTX-loaded NPs was also investigated in acidic and/or ROS conditions. As seen in Figure 2D, the DTX was completely released from the NPs under pH 5.0/1.0 mM H_2_O_2_ conditions, implying that the carrier was seriously disrupted under pH 5.0/H_2_O_2_ conditions, allowing the complete release of the DTX. Meanwhile, approximately 80% of the DTX was released from the NPs in pH 5.0 or 1.0 mM H_2_O_2_ medium within 48 h. However, only approximately 50% of the DTX was released from the NPs under pH 7.4. In addition, under the pH 5.0 and pH 5.0/H_2_O_2_ conditions, the CA was released from the NPs and completely released under the pH 5.0/1.0 mM H_2_O_2_ condition at 48 h. Incubation of NPs in PBS or 1.0 mM H_2_O_2_ resulted in minimal release of CA, suggesting that the pH-responsiveness of NPs comes from acetal cleavage between CA and α-CD (Figure S3). The result demonstrated that the NPs could achieve pH- and ROS-responsive drug release. Furthermore, the capability of dual-responsiveness of NPs can synergistically play significant strengthening effects in the treatment [44].

The effect of DTX-loaded NPs on intracellular H_2_O_2_ levels in 4T1 cells was evaluated. As shown in Figure S4, DTX/NPs and DTX/RGD NPs significantly increased the intracellular H_2_O_2_ concentration compared to the control group, free DTX and non-responsive PS NPs. The CA released from DTX/NPs and DTX/RGD NPs is known to elevate intracellular ROS through mitochondria dysfunction [45]. Moreover, DTX/RGD NPs induced more H_2_O_2_ generation than DTX/NPs, which might be attributable to the RGD-mediated endocytosis. As our previous study reported that ROS-responsive NPs depleted the H_2_O_2_ in 4T1 cells [42], the elevated H_2_O_2_ confirmed the release of CA from NPs in the acidic environment, which subsequently increased the intracellular H_2_O_2_ concentration. Furthermore, increased H_2_O_2_ would facilitate further release of the encapsulated DTX.

### 3.2. Cellular Uptake

**Figure 3:**
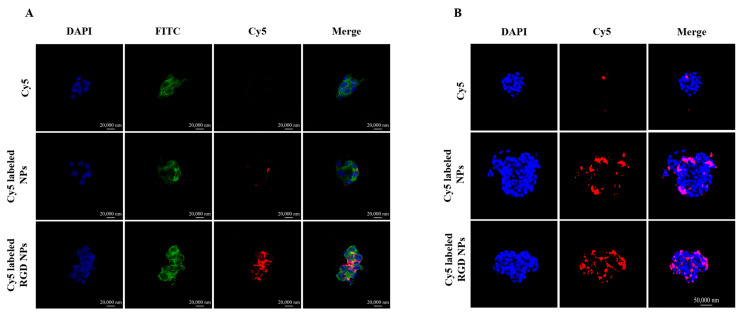
Cellular uptake of Cy5-labeled NPs in 4T1 cells and fluorescence distribution of Cy5-labeled NPs in 4T1 tumor spheroids. (**A**) The CLSM images of cellular uptake of Cy5-labeled NPs in 4T1 cells after 4 h treatment. Cell nuclei were stained with DAPI (blue), FITC-phalloidin for cytoskeleton staining (green) and Cy5-labeled NPs (red). Scale bar represents 20 μm. (**B**) Fluorescence distribution of Cy5-labeled NPs (red) in 4T1 tumor spheroids after 4 h treatment. Cell nuclei were stained with DAPI (blue) and Cy5-labeled NPs (red). Scale bar represents 50 μm.

Whether nanoparticles can be taken up by tumor cells is an important factor in determining their effectiveness in the body. We investigated the cellular uptake of Cy5-labeled NPs and RGD NPs in 4T1 cells. Figure 3A shows that the NPs were mainly distributed in the cytoplasm of the 4T1 cells. The fluorescence intensity of free Cy5 was extremely weak after 4 h of incubation, and the Cy5 fluorescence intensity of the NPs was significantly stronger than free Cy5. Meanwhile, compared to the NPs group, the RGD-functionalized NPs group showed a remarkably stronger fluorescence intensity in the 4T1 cells, indicating that RGD modification could enhance the uptake of NPs in 4T1 cells (Figure 3A). The cellular uptake was also quantified using flow cytometry, and the quantitative results also illustrated that the RGD-modified NPs more easily entered into 4T1 cells compared with NPs. Similarly, the internalized NPs in tumor cells were much higher than free drugs (Figure S5). These results demonstrated that the RGD modification promoted the cellular uptake of the NPs into α_V_β3-receptor-over-expressed cancer cells.

### 3.3. Three-Dimensional (3D) Tumor Spheroid Penetration of the NPs

The ability of the NPs to penetrate the tumor spheroids was investigated using CLSM with a layer-scanning mode. In Figure 3B, the Cy5 solution hardly penetrated into the interior of tumor spheroids and only weak fluorescence signals were observed around the spheroids. In comparison, the Cy5-labeled NPs and RGD NPs exhibited different levels of penetration into the cores of tumor spheroids. As shown in Figure 3B, both the Cy5-labeled NPs and the RGD NPs showed a remarkable penetration efficiency after 4 h of incubation compared with free Cy5, and the penetration efficiency was significantly increased after RGD NPs treatment compared with the NPs group. The optimal tumor penetration of RGD NPs was attributed to the high binding efficiency between the cRGD modified on the surface of NPs and αvβ3 receptors of tumor cells. These results confirmed that RGD-modified pH/ROS dual-responsive NPs had a satisfactory penetration efficiency into the tumor spheroids, which was beneficial to deliver the drug to the deep of tumor tissue and improve the antitumor effect.

### 3.4. In Vitro Cytotoxicity of the NPs on Tumor Cells

**Figure 4:**
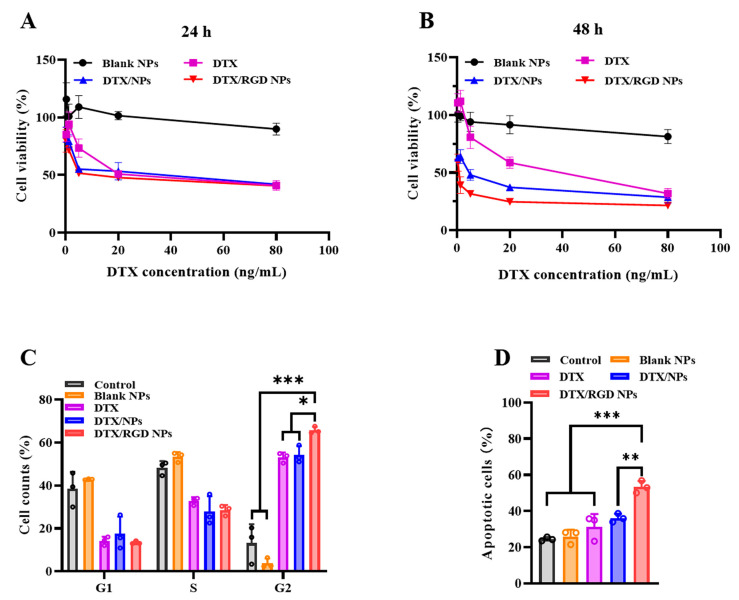
The in vitro antitumor activities of NPs against 4T1 cells. (**A**) Cell viabilities of 4T1 cells after treatment with blank NPs, DTX and DTX-loaded NPs at various drug concentration for 24 h. (**B**) Cell viabilities of 4T1 cells after treatment with blank NPs, DTX and DTX-loaded NPs at various drug concentrations for 48 h. (**C**) Quantitative analysis of cell-cycle profiles of 4T1 cells following treatment with blank NPs, DTX and DTX-loaded NPs for 48 h. (**D**) The apoptotic percentages of 4T1 cells following treatment with blank NPs, DTX and DTX-loaded NPs for 48 h. Each value represents the mean ± SD (*n* = 3). *, statistically different at *p* < 0.05; **, statistically different at *p* < 0.01; ***, statistically different at *p* < 0.001, compared with DTX/RGD NPs.

The in vitro cytotoxicity of the DTX-loaded NPs on 4T1 cells was evaluated with a CCK-8 assay. Compared with the blank NPs, the DTX and DTX-loaded NP groups significantly decreased the viability of the 4T1 cells with increasing drug concentrations and exhibited strong antitumor activity after 24 h treatment (Figure 4A). When the concentration of DTX was lower than 20 ng/mL, the antitumor effect of DTX-loaded NPs was significantly stronger than that of DTX. The IC_50_ of DTX, NPs and RGD NPs was 32.13 ng/mL, 24.87 ng/mL and 15.57 ng/mL, respectively. The IC_50_ of RGD NPs was lower than that of the DTX and the NPs, implying that the RGD NPs had the strongest antitumor effect among groups. After 48 h of treatment, the RGD NPs still exhibited the strongest cytotoxicity against the 4T1 cells (Figure 4B). Accordingly, the IC_50_ value of RGD NPs was 0.55 ng/mL, and the IC_50_ of DTX and NPs was approximately 58-fold or seven-fold, respectively, than RGD NPs, which demonstrated that RGD-modified NPs significantly improved the antitumor activity of DTX compared with the free DTX and unmodified NPs. Similar to 4T1 cells, the RGD NPs also had the strongest antitumor effect on MDA-MB-231 cells compared to other groups (Figure S6).

### 3.5. Cell-Cycle Assay

DTX has been demonstrated as a cell cycle-specific antitumor drug, acting mainly in the G2/M phase of tumor cells [28]. As shown in Figure 4C, compared with the control and blank NPs, there was a significantly increased number of cells in the G2/M phase after treatment with DTX and DTX-loaded NPs, implying that DTX and DTX-loaded NPs promoted the arrest of the cell cycle at the G2/M phase. It was worth noting that the cell proportion at the G2/M phase of cells treated with RGD-modified NPs showed an evident increase compared to DTX and unmodified NPs, demonstrating that the RGD-modified NPs had the strongest blocking effect on the 4T1 cells’ cycle compared to other groups. The result can be explained by the RGD modification improving the cellular uptake of NPs and then more NPs in the cells releasing more DTX to inhibit the proliferation of tumor cells and enhance the antitumor effect.

### 3.6. Cell Apoptosis

The antitumor effect of DTX-loaded NPs was further verified with an Annexin V-FITC Apoptosis Detection Kit. As shown in Figure 4D, although there was no significant difference in the apoptotic ratio between the NP and DTX groups, NPs showed significantly higher cell apoptotic percentages compared to the control and blank NPs. With regard to the RGD NP-treated groups, significantly higher apoptotic cell percentages were observed compared to other groups. These results indicate that both DTX and its nanoformulations could induce the apoptosis of 4T1 cells, and RGD-modified NPs showed an obviously increased antitumor activity compared to free DTX and unmodified NPs. This result was consistent with the in vitro cytotoxicity results.

### 3.7. In Vivo Biodistribution

**Figure 5:**
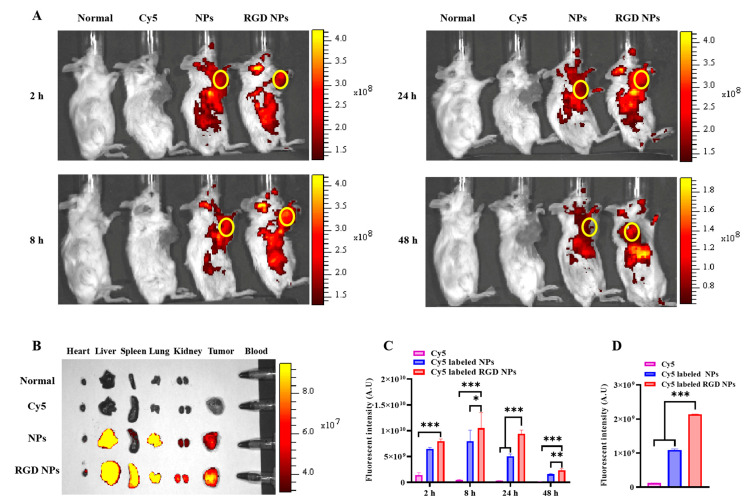
In vivo biodistribution of free Cy5, Cy5-labeled NPs and Cy5-labeled RGD NPs in 4T1 tumor-bearing mice. (**A**) In vivo fluorescence images of mice 2 h, 8 h, 24 h and 48 h after administration. Normal mice were injected with saline. (**B**) Ex vivo fluorescence image of the excised major tissues and tumors at 48 h post-injection. (**C**) Semi-quantitative analysis of fluorescence intensity in tumor tissues at the indicated time points. (**D**) Semi-quantitative analysis of fluorescence intensity of tumors excised from mice. *, statistically different at *p* < 0.05; **, statistically different at *p* < 0.01; ***, statistically different at *p* < 0.001, compared with Cy5-labeled RGD NPs.

The effective accumulation of NPs at the tumor site is an important factor in determining their efficacy; thus, we investigated the tumor-targeting capability of the NPs in a 4T1 tumor-bearing mouse model. As shown in Figure 5A, both the Cy5-labeled NPs and the RGD NPs showed stronger fluorescence signals in tumor sites than free Cy5 at the observed time, indicating that Cy5 was rapidly cleared by the body and that there was an obvious tumor-targeting effect of Cy5-labeled NPs and RGD NPs. More importantly, the Cy5-labeled RGD NPs exhibited stronger fluorescence signals at the tumor site after treatment for 8 h, 24 h and 48 h than the Cy5-labeled NPs group (Figure 5A). The ex vivo images further confirmed that RGD-modified NPs significantly enhanced the tumor-targeting capacity of the NPs compared to unmodified NPs after 48 h of treatment (Figure 5B). The semi-quantitative results of images in vivo and ex vivo similarly confirmed that the RGD modification significantly enhanced the tumor targeting of the NPs (Figure 5C,D). In addition, the images of the frozen sections of the tumor tissue in different groups further demonstrated that the RGD NPs had a satisfactory tumor-targeting ability. The Cy5 signal in RGD NPs was significantly higher than that of non-targeted NPs and free Cy5 (Figure S7). Taken together, these results demonstrate that RGD-modified NPs could effectively target the tumor regions and accumulate over the long term, which is beneficial to improve the specificity and targeted therapeutic efficacy of chemotherapeutic drugs.

### 3.8. The Evaluation of Antitumor Effect of NPs In Vivo

**Figure 6:**
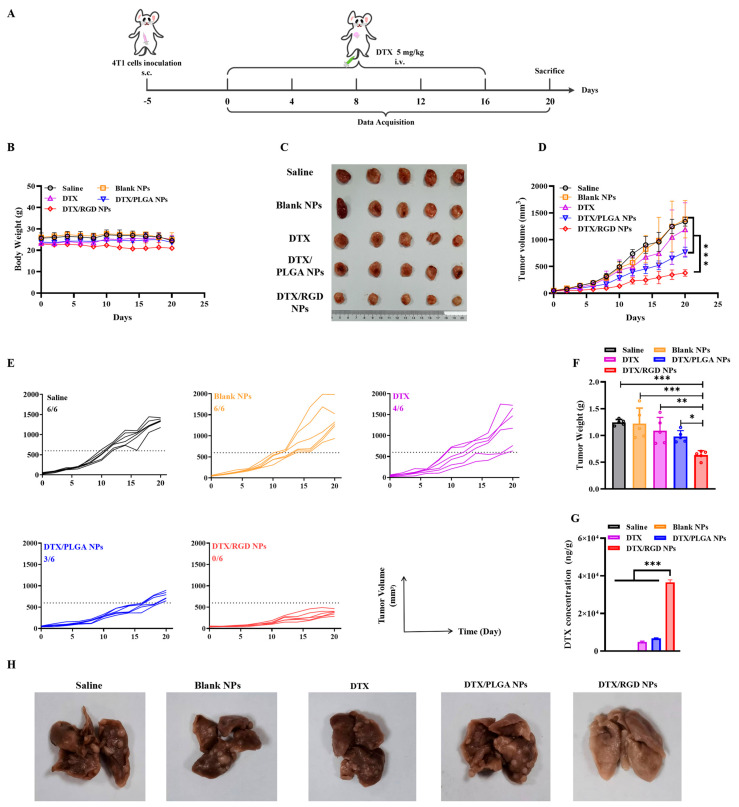
In vivo antitumor efficacy evaluation of blank NPs, DTX and various DTX-loaded NPs in 4T1 tumor-bearing mice. (**A**) The administration time after tumor inoculation. Mice received 5 mg/kg of DTX or 5 mg/kg of various DTX-loaded NPs via vein every four days for five times. (**B**) The body weight changes of mice following different treatment (*n* = 6). (**C**) Representative photographs of tumor tissues of mice following different treatment. (**D**) The tumor growth curves of mice following different treatment (*n* = 6). (**E**) Individual tumor volumes of mice following different treatments. The ratio refers to the proportion of mice with tumors that exceed 600 mm^3^ on day 20. (**F**) The tumor weight of mice following different treatments. (**G**) The DTX concentration in tumor tissues of mice following different treatments (*n* = 3). (**H**) The photo images of collected lungs in the mice following different treatments on day 20. *, statistically different at *p* < 0.05; **, statistically different at *p* < 0.01; ***, statistically different at *p* < 0.001, compared with DTX/RGD NPs.

Our previous study demonstrated that RGD-modified NPs had the optimal antitumor effect in vitro and tumor-cell targeting in vivo; thus, the in vivo antitumor efficacy of cRGD-modified NPs was further evaluated in 4T1 tumor-bearing mice. As shown in Figure 6B, all tumor-bearing mice had no obvious changes in body weight during the therapy. Tumor growth inhibition was observed in all drug-treated groups compared with the saline and blank NP groups. DTX slightly inhibited the growth of the primary tumor but did not show a significant antitumor effect, which may be explained by the fact that small-molecule chemotherapeutic agents are easily eliminated by the body and hardly enter the interior of the tumor. Differently, the DTX-loaded NPs significantly improved the antitumor efficacy compared to the DTX group, mainly due to the well-known EPR effect of NPs. The DTX-loaded PLGA NPs reduced by 35% of the tumor volume relative to DTX at day 20. More importantly, the DTX-loaded RGD NPs remarkably inhibited tumor growth compared to that of non-responsive PLGA NPs (Figure 6C,D). At day 20, the proportions of tumors exceeding 600 mm^3^ in all groups were 6/6 (saline), 6/6 (blank NPs), 4/6 (DTX), 3/6 (DTX/PLGA NPs) and 0/6 (DTX/RGD NPs), respectively. Accordingly, the proportion of tumors exceeding 600 mm^3^ in the group of DTX/RGD NPs remained the lowest in all groups, showing the optimal therapeutic efficacy of DTX/RGD NPs (Figure 6E). The results for tumor weight were consistent with those for tumor volume and also demonstrated the significant tumor-inhibition efficiency of DTX/RGD NPs in vivo (Figure 6F). To further validate our experimental results, we further examined the DTX content in the tumor tissues of each group in mice. As shown in Figure 6G, DTX was barely detectable in the saline and blank NP groups. A certain amount of DTX could be detected in the DTX and PLGA NP groups. It is worth noting that the concentration of DTX in the RGD NP group was much higher than in the DTX and PLGA NP groups. This result further supports the in vivo biodistribution assays. In addition, we investigated the capability of DTX/RGD NPs to inhibit lung metastasis of breast cancer. As shown in Figure 6H and Figure S8, a large number of metastatic nodules was observed in the lung tissue of the mice after treatment with saline and blank NPs. Compared to the saline and blank NP groups, DTX and PLGA NP treatment obviously blocked the lung metastasis. Notably, there were no metastatic clots observed in the lung tissue of mice after DTX/RGD NP treatment. These results indicate that DTX/RGD NPs enhanced the antitumor efficacy and antimetastatic effect of DTX in vivo.

## 4. Conclusions

In summary, we reported a cyclic RGD-functionalized pH/ROS-responsive NP for enhanced cancer therapy. The NPs displayed pH/ROS dual-responsiveness and complete drug release in vitro. Moreover, the NPs could effectively target 4T1 cells and enter inside tumor spheroids. In addition, the NPs greatly inhibited the proliferation and induced the apoptosis of tumor cells in vitro. In vivo, DTX/RGD NPs actively accumulated at the tumor site via an α_V_β3-mediated active-targeting effect and efficiently inhibited 4T1 tumor growth and lung metastasis. Overall, our study provides a great potential strategy for the treatment of metastatic breast cancer and clinical translation.

## Data Availability

Not applicable.

## Supplementary Materials

The following supporting information can be downloaded at: https://www.mdpi.com/article/10.3390/pharmaceutics15071827/s1, Scheme S1: Synthetic route of CA-Oxi-αCD. Scheme S2: Illustration of dual-responsive behaviors of CA-Oxi-αCD carrier in ROS/acidic environment. Table S1: Physicochemical properties of various nanoformulations. Figure S1: ^1^H NMR spectrum of CA-Oxi-αCD carrier. Figure S2: The size changes of NPs in various media. Figure S3: The in vitro drug-release behavior of CA from NPs in various release media within 48 h. Figure S4: The intracellular concentration of H_2_O_2_ in 4T1 cells after treatment with DTX, PS NPs (non-ROS-responsive) and DTX-loaded NPs. Figure S5: The cellular uptake of 4T1 cells treated with Cy5, Cy5-labeled NPs and Cy5-labeled RGD NPs for 4 h. Figure S6: Cell viabilities of MDA-MB-231 cells after treatment with blank NPs, DTX and DTX-loaded NPs at various drug concentrations for 72 h. Figure S7: The CLSM images and semi-quantitative analysis of fluorescence intensity in CLSM images of tumor tissues from mice after treatment with Cy5, Cy5-labeled NPs and Cy5-labeled RGD NPs in vein for 48 h. Figure S8: The histopathologic examination of the major tissues from mice in different groups with H&E staining.
