# Supplementary material for: Cyclic RGD-Functionalized pH/ROS Dual-Responsive Nanoparticle for Targeted Breast Cancer Therapy
Source: Pharmaceutics. 2023 Jun 26;15(7):1827. doi: 10.3390/pharmaceutics15071827 (PMC10386338; doi:10.3390/pharmaceutics15071827)
Supplement: Supplementary file 1 [file pharmaceutics-15-01827-s001.zip › pharmaceutics-2427346-supplementary.pdf]

Supporting Information

Cyclic RGD-functionalized pH/ROS dual-responsive nanoparticle for targeted breast cancer therapy

Pu Yao[a,†], Xiaowen Wang[a,†], Qianmei Wang[a,†], Qing Dai[a], Yu Peng[b], Qian Yuan[a], Nan Mou[a], Shan Lv[a], Bangbi Weng[a], Yu Wang[a,*], Fengjun Sun[a,*]

a Department of Pharmacy, Southwest Hospital, Army Medical University (Third Military Medical University), Chongqing 400038, China

b Department of Oncology, Southwest Hospital, Army Medical University (Third Military Medical University), Chongqing 400038, China

*Corresponding authors:

Yu Wang, MD, Associate Prof.

Department of Pharmacy,

Southwest Hospital,

Army Medical University (Third Military Medical University), Chongqing 400038, China

E-mail: wy5855112@163.com

Fengjun Sun, PhD, Associate Prof.

Department of Pharmacy,

Southwest Hospital,

Army Medical University (Third Military Medical University), Chongqing 400038, China

E-mail: fengj_sun@163.com

ORCID: 0000-0003-2679-8797

[†] These authors contributed equally to this work.

Reagents

All chemical reagents and anhydrous solvents are obtained from commercial sources and used directly upon receipt. 4-dimethylaminopyridine (DMAP), trimethoxymethane, α-Cyclodextrin (α-CD), N,N'-Carbonyldiimidazole (CDI), anhydrous dimethyl sulfoxide (DMSO) were purchased from Aladdin (Shanghai, China). Cinnamaldehyde, Pyridin-1-ium 4-methylbenzenesulfonate (PPTs) were purchased from *J&K* Scientific Co., Ltd. (Beijing, China). 4-Hydroxymethylphenylboronic acid pinacol ester was purchased from Sigma-Aldrich (St. Louis, U.S.A.). Cy5 free acid and Cy5-NHS ester were purchased from Ruixi Biological Technology Co., Ltd. (Xi'an, China).

Materials synthesis

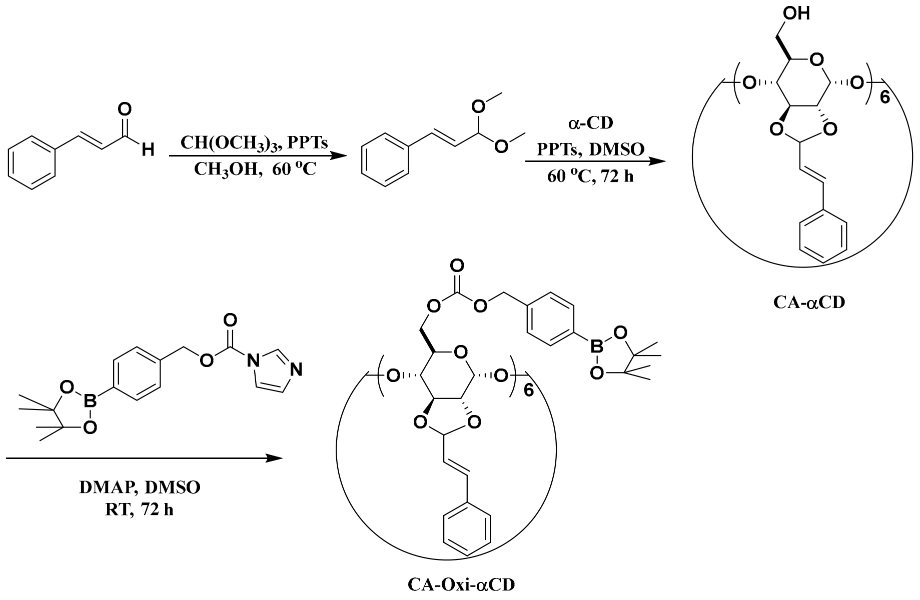

Scheme S1. Synthetic route of CA-Oxi-αCD.

Synthesis of compound 1

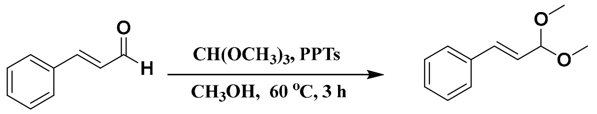

1

Cinnamaldehyde (CA, 5.3129 g, 40.20 mmol) and trimethoxymethane (19.13 g, 180.90 mmol) were dissolved in methanol (50 mL), then Pyridin-1-ium 4-methylbenzenesulfonate (PPTs, 2.0180 g, 8.01 mmol) was added, then the reaction was refluxed at 60 °C and monitored by thin layer chromatography (TLC, petroleum ether:ethyl acetate = 20:1). With a reaction time of 3 h, TLC indicated that the reaction was completed, saturated $NaHCO_3$ (20 mL) was added to quench the reaction, such a mixture was submitted to stir at room temperature for 10 min, followed by the extraction with ethyl acetate (40 mL×3). All ethyl acetate was combined and dried with $MgSO_4$ for at least 2 h under moderate stirring. The ethyl acetate was evaporated by rotary evaporator to afford the product CA acetal (compound **1**, 6.8342 g). The reaction residue was used in

the next step without further purification.

Synthesis of compound 2

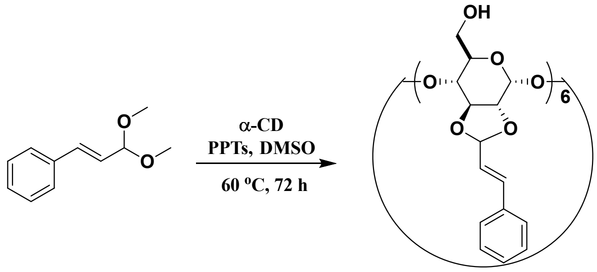

CA-αCD

1 2

α-cyclodextrin (α-CD, 1.6293 g) and CA acetal (compound **1**, 3.5821 g) were dissolved in anhydrous DMSO (13 mL), then pyridine-1-4-methylbenzylammonium sulfonate (PPTs, 0.2522 g) was added to the solution. After degassed with three consecutive vacuum/argon-fill cycles, the resulting mixture was stirred at 60 °C for 72 h. The reaction solution was poured into acetone (130 mL) to precipitate the crude product, then filtered through reduced pressure and rinsed by acetone (20 mL×3), dried at 80 °C to provide the compound **2** as light yellow solid (CA-αCD, 2.0342 g).

Synthesis of compound 3

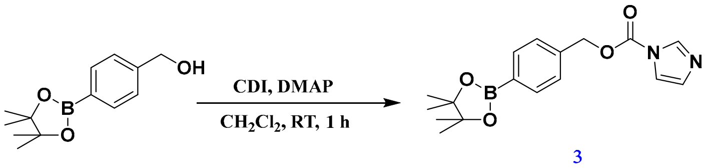

3

4-Hydroxymethylphenylboronic acid pinacol ester (5.4300g, 23.19 mmol) was dissolved in anhydrous CH_2Cl_2 (36 mL) and then N,N'-Carbonyldiimidazole (CDI, 7.5222 g, 46.39 mmol) was added, such a mixture was stirred at room temperature with a reaction time of 1 h, after which another CH_2Cl_2 (36 mL) was added to the reaction mixture, the solution was washed with DI water (30 mL ×3) to remove superfluous CDI, then further washed with saturated NaCl (30 mL), the residual CH_2Cl_2 was dried with $MgSO_4$ for at least 2 h under moderate stirring. The CH_2Cl_2 was evaporated under reduced pressure to provide the desired compound **3** as white solid (7.6321 g).

Synthesis of compound 4

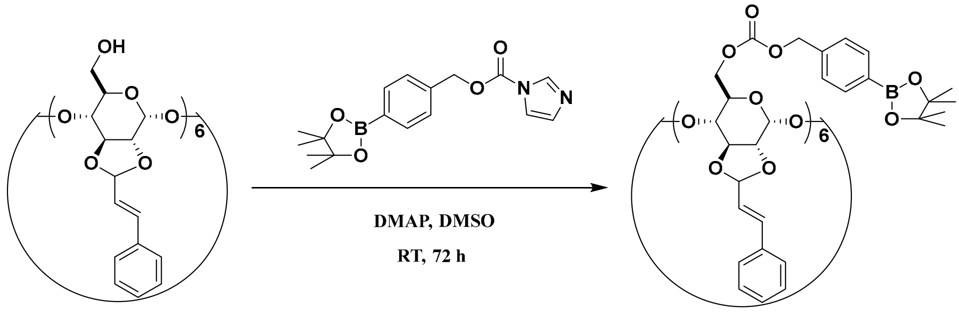

CA-αCD **2** CA-Oxi-αCD **4**

Compound **2** (0.9438 g) was dissolved in anhydrous DMSO (30 mL), then DMAP (2.0867 g) and CDI-activated pinacol ester (compound **3**, 1.1211 g) were introduced to the solution. The round-bottom was submitted to degassed with three consecutive vacuum/argon-fill cycles, then the resulting mixture was stirred at 30 ºC for 72 h. After the reaction was completed, the crude product was precipitated from 30 mL water and collected by centrifugation, the obtained solid was dialyzed for 48 h in DI water (MWCO: 3.5 kDa) upon stirring for purification. Finally pure compound **4** was gained as off-white solid by lyophilization (CA-Oxi-αCD, 0.5634 g).

Characterization

^{1}H Nuclear Magnetic Resonance (^{1}H NMR) data was collected at 25 °C on a 600 MHz spectrometer (Direct Drive 2, Agilent) and chemical shifts were reported relative to tetramethylsilane (TMS, δ = 0).

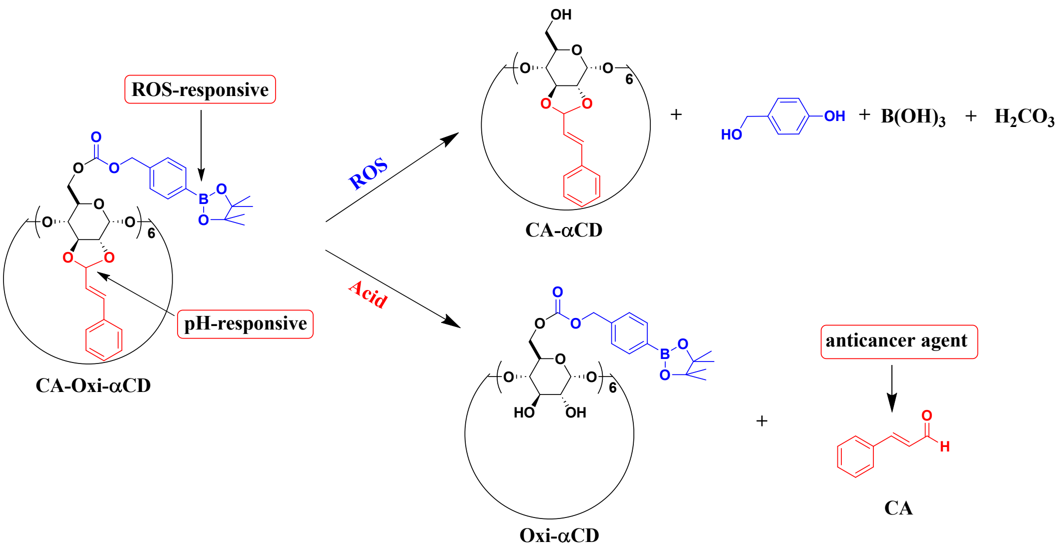

Scheme S2. Illustration of dual-responsive behaviors of CA-Oxi-αCD carrier in ROS/acidic environment.

Table S1. Physicochemical properties of various nanoformulations.

Nanoformulations	Size (nm)	PDI	Zeta potential (mV)	Drug loading (w/w %)	Encapsulation efficiency (%)
Blank NPs	228.67 ± 2.07	0.16 ± 0.03	-16.77 ± 0.94	-	-
DTX/NPs	228.73 ± 7.57	0.14 ± 0.05	-16.6 ± 1.51	18.32 ± 3.70	60.56 ± 1.17
DTX/RGD NPs	217.00 ± 2.70	0.16 ± 0.04	-19.37 ± 0.58	19.37 ± 3.05	73.32 ± 5.04

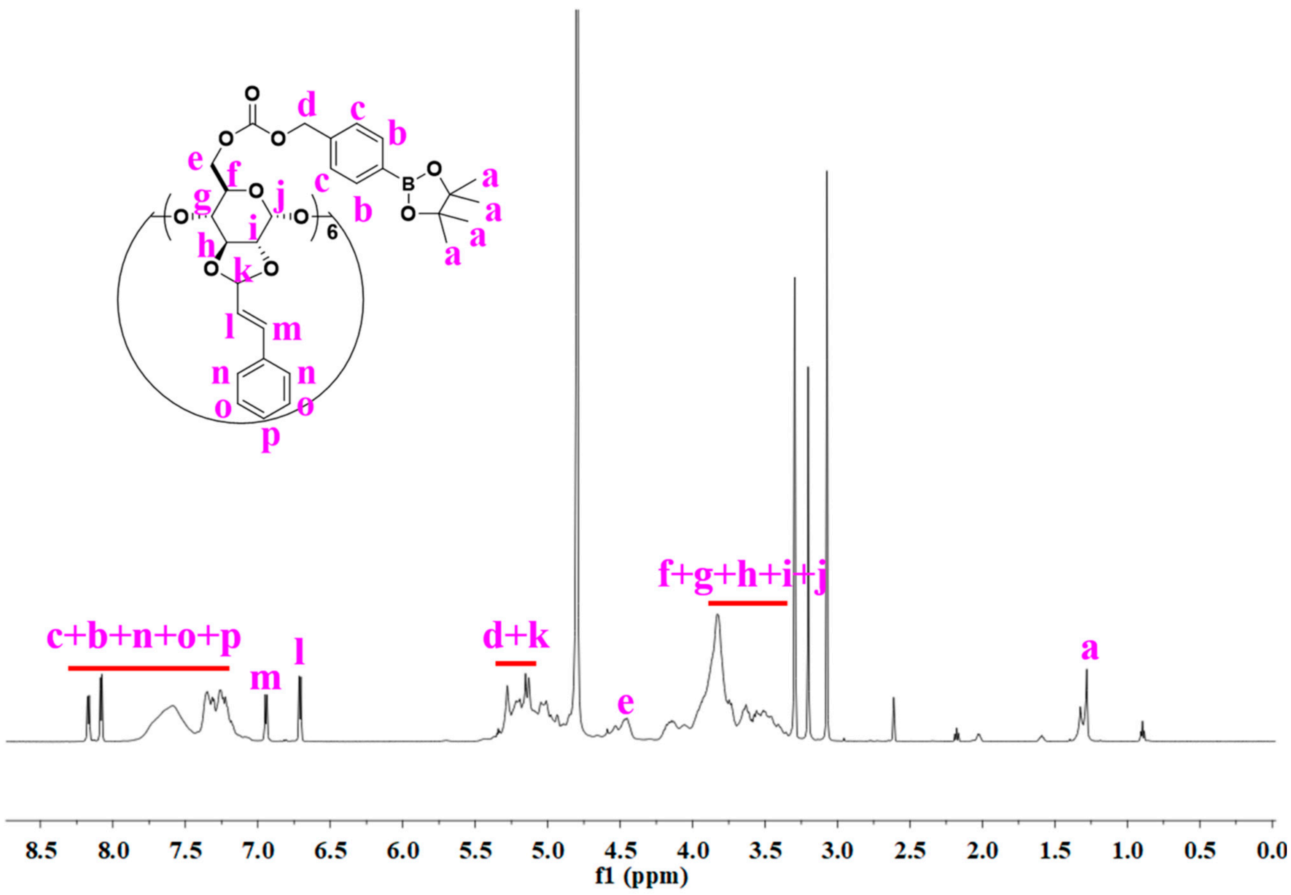

Figure S1. ^{1}H NMR spectrum of CA-Oxi-αCD carrier.

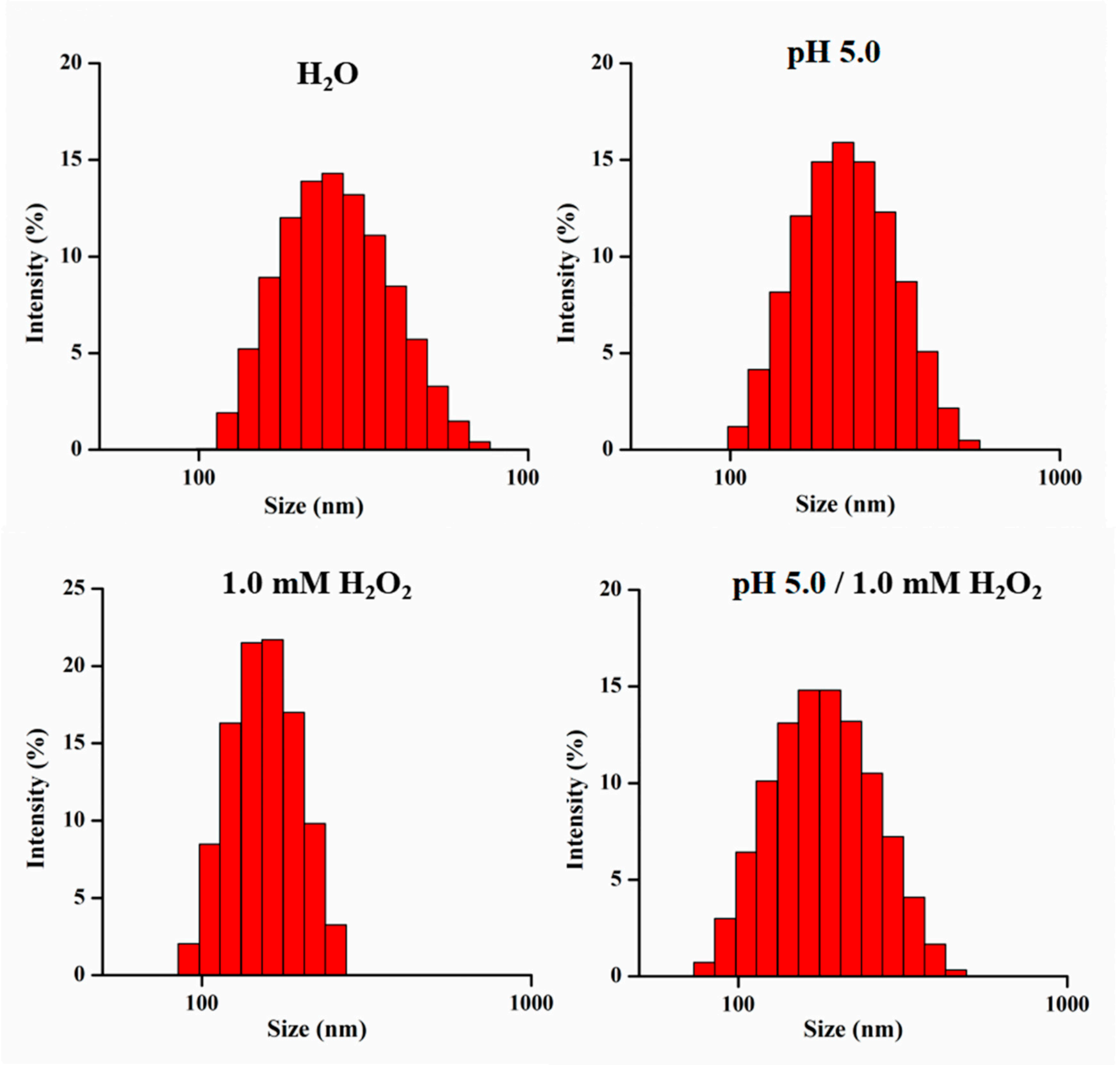

Figure S2. The size changes of NPs in various medium.

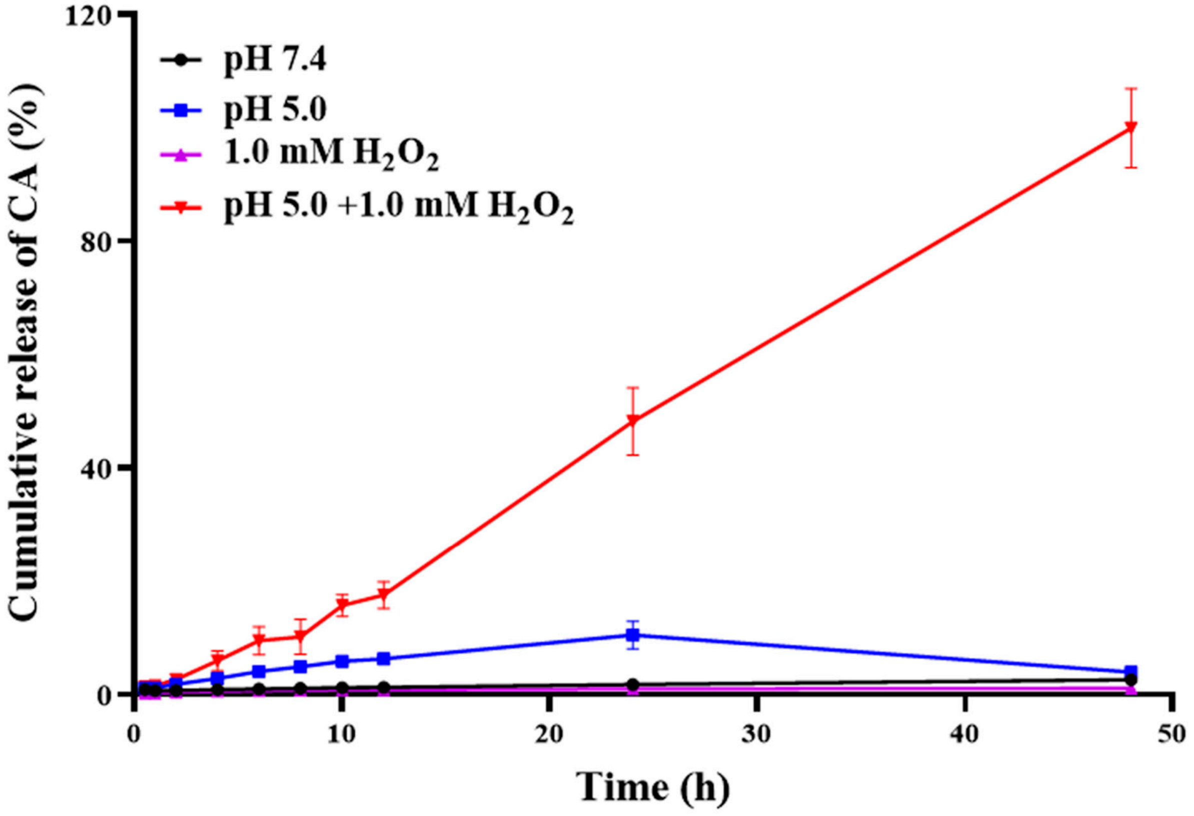

Figure S3. The *in vitro* drug release behavior of CA from NPs in various release medium within 48 h. Each value represents the mean ± SD (n =3).

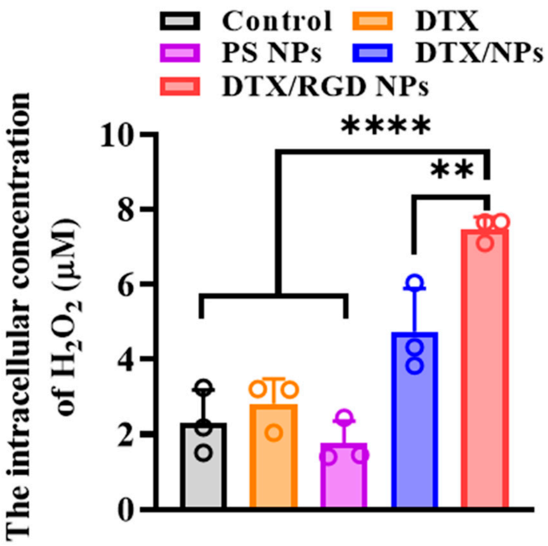

Figure S4. The intracellular concentration of H_2O_2 in 4T1 cells after treatment with DTX, PS NPs (non ROS- responsive) and DTX loaded NPs. Cells treated with cell culture medium as control. Each value represents the mean ± SD (n =3). *, statistically different at $P < 0.05$, **, statistically different at $P < 0.01$, ***, statistically different at $P < 0.001$, ****, statistically different at $P < 0.0001$, compared with DTX/RGD NPs.

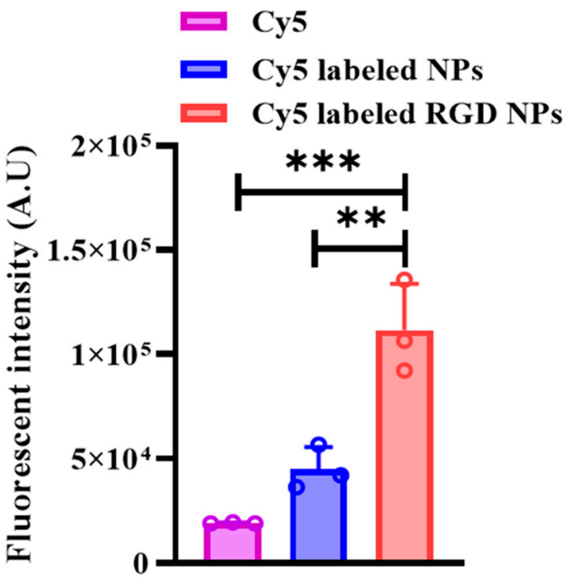

Figure S5. The cellular uptake of 4T1 cells treated with Cy5, Cy5-labeled NPs, and Cy5-labeled RGD NPs for 4 h. *, statistically different at $P < 0.05$, **, statistically different at $P < 0.01$, ***, statistically different at $P < 0.001$, compared with Cy5-labeled RGD NPs (n=3).

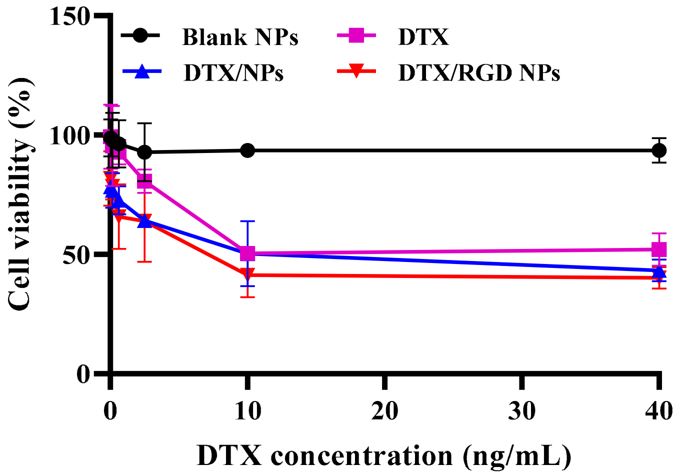

Figure S6. Cell viabilities of MDA-MB-231 cells after treatment with blank NPs, DTX, and DTX loaded NPs at various drug concentration for 72 h. Each value represents the mean ± SD (n =3).

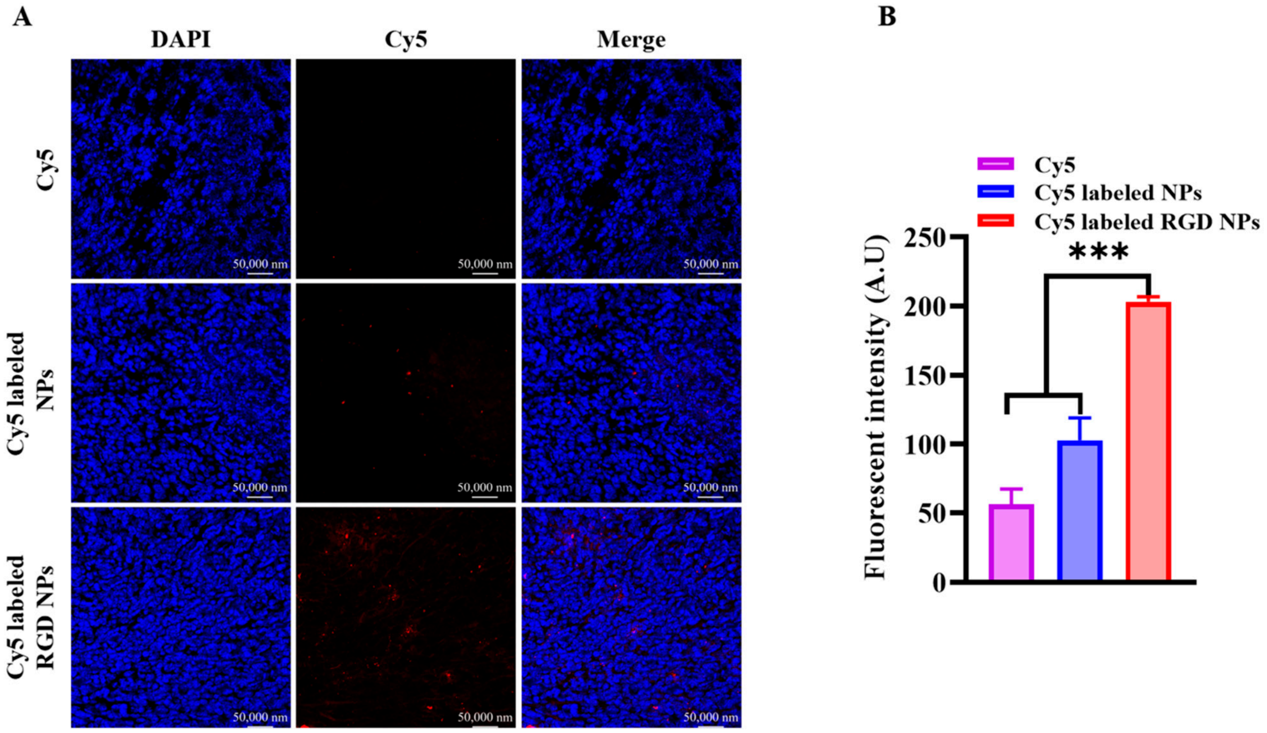

Figure S7. (A) The CLSM images of tumor tissues from mice after treated with Cy5, Cy5-labeled NPs, and Cy5-labeled RGD NPs in vein for 48 h. Blue, cell nuclei stained with DAPI. Red, Cy5-labeled NPs, Scale bar represents 50 μm. (B) Semi-quantitative analysis of fluorescence intensity in CLSM images of tumor tissues at 48 h. *, statistically different at $P < 0.05$, **, statistically different at $P < 0.01$, ***, statistically different at $P < 0.001$, compared with Cy5-labeled RGD NPs (n=3).

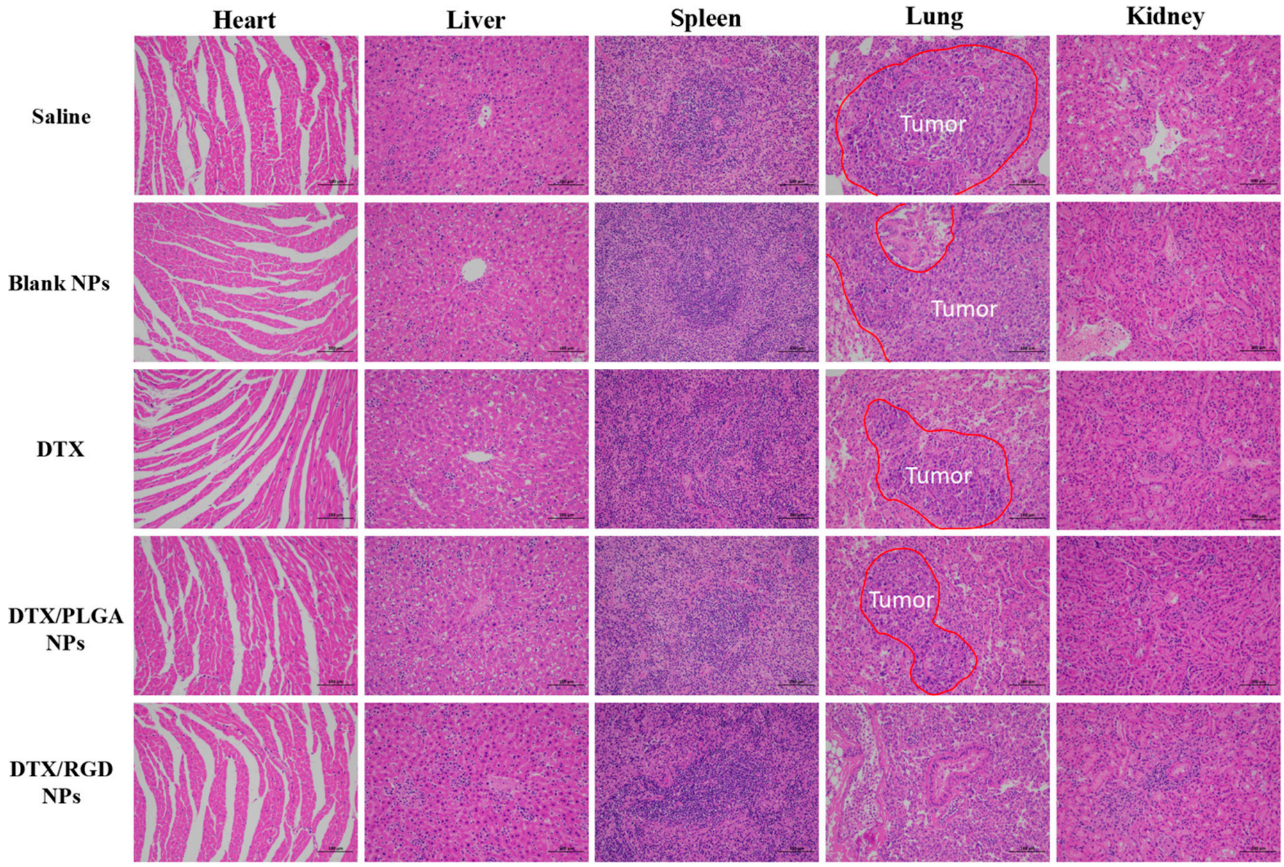

Figure S8. The histopathologic examination of the major tissues from mice in different groups by H&E staining. Pulmonary metastatic nodules are indicated within the red markers. Scale bar represents 100 μm.
